# Development of Time Sensitivity and Information Processing Speed

**DOI:** 10.1371/journal.pone.0071424

**Published:** 2013-08-21

**Authors:** Sylvie Droit-Volet, Pierre S. Zélanti

**Affiliations:** Laboratoire de Psychologie Sociale et Cognitive, Centre national de la recherche scientifique, Université Blaise Pascal, Clermont-Ferrand, France; National University of Singapore, Singapore

## Abstract

The aim of this study was to examine whether age-related changes in the speed of information processing are the best predictors of the increase in sensitivity to time throughout childhood. Children aged 5 and 8 years old, as well adults, were given two temporal bisection tasks, one with short (0.5/1-s) and the other with longer (4/8-s) anchor durations. In addition, the participants' scores on different neuropsychological tests assessing both information processing speed and other dimensions of cognitive control (short-term memory, working memory, selective attention) were calculated. The results showed that the best predictor of individual variances in sensitivity to time was information processing speed, although working memory also accounted for some of the individual differences in time sensitivity, albeit to a lesser extent. In sum, the faster the information processing speed of the participants, the higher their sensitivity to time was. These results are discussed in the light of the idea that the development of temporal capacities has its roots in the maturation of the dynamic functioning of the brain.

## Introduction

From the moment of their birth, children are immersed in time. Every day, they experience the timing and duration of actions and events and rapidly learn to monitor time and evaluate its passage. Evidence of children's early timing abilities has been provided by studies conducted among infants and 3-year-old children that have shown that they accurately discriminate different stimulus durations [1]–[5]. In addition, their timing behavior obeys Weber's law. More precisely, the variability (Standard Deviation) in their temporal discrimination increases linearly with the mean of the durations for discrimination. Their timing behavior therefore strongly suggests that the timing observed in human adults and other animals is functional in these young subjects at an early age.

Nevertheless, the capacity to judge time precisely in a wide variety of temporal tasks (temporal production, reproduction, generalization and bisection) improves during childhood [3], [6]–[12]. In their attempt to trace the typical development of temporal discrimination capacities, researchers have employed the temporal bisection task [13]. This task is particularly suitable for use with very young children and has also been used in lower animals, such as rats. In this task, children are presented with a short (*S*) and a long (*L*) standard duration. They are then presented with comparison durations (*D*) (equal to *S* or *L* or of intermediate values) and must judge whether these durations are more similar to *S* or *L*. In this bisection task, children of different ages exhibit orderly psychophysical functions, with the proportion of long responses (*p*(long)) increasing with the comparison duration values, thus revealing their ability to discriminate time. The point of subjective equality (*D*(p(long)  = .50), also called the bisection point (BP), appears to be similar across ages. However, the psychometric function slopes are systematically flatter in younger children. When indexes of variability in time discrimination are calculated, such as the Difference Limen (DL) (*D*(*p*(long)  = .75–*D*(*p*(long)  = .25)/2) or the Weber Ratio (WR) – which is a type of coefficient of variation (DL/BP) -, their values decrease with increasing age. This demonstrates that time sensitivity in a temporal discrimination task, such as the bisection task, improves during childhood.

In sum, beyond similarities across ages in the basic functioning of temporal discrimination, there is also an age-related improvement in sensitivity to time. The question is: what determines these age-related changes in time sensitivity? Recent neuroscientific studies have revealed that the processing of time involves distributed areas in the brain (cerebellum, supplementary motor areas, prefrontal and parietal cortex, caudate and putamen and the right insula), although the fronto-striatal system seems to be the key cerebral structure responsible for the encoding of time across different time-related tasks [14]–[17]. It is thus logical to assume that the developmental improvement in time sensitivity results from the maturation of these neural circuits, although the impact of experience cannot be excluded. Indeed, the increase in white matter during childhood and adolescence, as well as the elimination and pruning of neural processes (i.e. axonal and dendritic processes), may increase the speed and efficiency of neural transmission, as well as the connectivity between different brain regions [18], [19]. However, the fronto-striatal system contributes to the executive functions that are involved in performing not only temporal but also other cognitive tasks [20], [21]. Consequently changes in time sensitivity may arise from the development of general functions that operate across a wide range of different cognitive processes rather than from a time-specific mechanism.

In order to examine the extent to which the development of general cognitive functions contributes to the improvement of time sensitivity in healthy children, we conducted a series of studies using the temporal bisection task in children aged between 5 and 9 years whose cognitive abilities had been assessed using various neuropsychological tests [22]–[24]. The results of these studies indicated that two cognitive dimensions are able to account for the majority of developmental changes in sensitivity to time: (1) selective attention and (2) working memory. Selective attention refers to the controlled processes that enable individuals to focus attention on relevant information while shutting out interfering irrelevant information. Zélanti and Droit-Volet assessed this function in children by means of the visual subtest of the developmental neuropsychological assessment (Nepsy) [25] which measures selective attention with an inhibitory component [22]. Working memory refers to processes that enable individuals to keep information active while integrating it with other information until the problem at hand is solved. A frequently used measure is the maximum amount of information that can be repeated backward (e.g., visuo-spatial movements in the Corsi test). However, although these two cognitive dimensions draw on partially separable processes, they are closely related. According to Baddeley's model, working memory consists of storage systems as well as of a central executive control system that plays the role of “attentional controller” responsible for the control and regulation of cognitive resources [26]–[28]. As both selective attention and working memory are based on the management of limited attentional resources, Droit-Volet argued that the most important sources involved in the typical development of time abilities arise from the development of attentional capacities rather then from the development of a specific mechanism devoted to time [29]. This view is consistent with the results of numerous studies showing that adults and children with ADHD, a disorder characterized by attention and working memory deficits, perform poorly in various temporal tasks [30].

However, in Zélanti and Droit-Volet's studies conducted in healthy children, the proportion of individual variance in time sensitivity explained by scores on selective attention and working memory tests was relatively low [22]–[24]. In analyses performed by these authors, age was always shown to be a reliable predictor of inter-individual variances in the development of temporal performance. This suggests that changes in cognitive dimensions other than selective attention and working memory might also contribute to the increase in the efficiency of time processing throughout childhood. Studies conducted in the field of cognitive development have shown a high correlation between different cognitive dimensions involved in the executive control of cognition. Nevertheless, models of the development of intelligence suggest that cognitive development results from a cascade of related processes in which age-related changes in the speed of information processing play a critical role [31]–[34]. According to Demetriou et al., the changes in information processing speed would be “followed in time” by changes in working memory and selective attention [31]. Kail has indeed demonstrated that the development of information processing speed mediates the development of working memory capacities [35]–[36]. Age-related changes in the speed of processing of the ongoing information flow might indeed make it possible to protect the system from intrusive irrelevant information and increase the working memory space available for the storage and utilization of relevant information. In other words, when information is processed faster, it is less vulnerable to interference and available working memory capacity increases. As argued by Luna, Garver, Urban, Lazar and Sweeney, “developmental improvements in processing speed may reflect the overall benefit of increase of efficiency of neural communication associated with increase of myelination, whereas development of inhibition and working memory may reflect the increased efficiency of particular brain regions associated with local refinements in brain circuits, reflecting changes in synaptic organization” (p. 2369) [37]. Although cognitive processes interact and overlap, the speed of information processing may thus play a specific basic role in controlling cognition, especially during the processing of dynamic information such as time. As we discuss below, the encoding and judgment of time are inherent to the dynamic functioning of the brain. The efficiency of temporal information processing may therefore be linked to accelerated information processing. In other words, the faster information processing is, the more sensitive subjects would be to time. As far as the development of time processing is concerned, it can therefore be assumed that the increase in speed of information processing during childhood and adolescence should be one of the best predictors of developmental changes in time sensitivity.

In the present study, children aged 5 and 8 years, as well as adults, were given two temporal bisection tasks, one with short anchor durations (0.5/1-s) and the other with longer anchor durations (4/8-s) whose processing is thought to require more attention [20], [38]. In addition, the participants' cognitive abilities in terms of short-term memory, working memory, selective attention and information processing speed were assessed using different neuropsychological tests. We hypothesized that the speed of information processing would be a better predictor of age differences in sensitivity to time than working memory or selective attention.

## Materials and Methods

### Participants

The sample was composed of 180 participants (91 girls and 89 boys): 60 aged 5 years (mean age  = 5.76, SD  = 0.31, 22 girls and 38 boys), 60 aged 8 years (mean age  = 8.82, SD  = 0.44, 34 girls and 26 boys) and 60 adults (mean age  = 23.97, SD  = 2.79, 35 women and 25 men). The children were recruited from nursery and primary schools at St Germain des Fossés and the adults were undergraduate psychology students at Clermont-Ferrand, all in the Auvergne region of France. The students and the children's parents signed a written consent. The inspector of the academy (local representative of the French Education Ministery), and the directors of schools also signed a formal agreement to conduct this study with the children in their school. This experiment conducted in typical children and students in 2011–2012 has been approved by Clermont-Ferrand Sud-Est VI Statutory Ethics Committee (Comité de Protection des Personnes (CPP) Sud-Est 6, France) according to the articles of law L. 1121–1–1 and R 1121–3.

### Material

The children performed the test individually in a quiet room at their schools, while the adults completed the test at the university. In the bisection task, the stimulus to be timed was a blue circle (6 cm in diameter) presented in the center of the screen of a PC computer using E-prime software. The participants gave their responses (short or long) orally and the experimenter recorded these responses by pressing the *K* and *D* keys on the computer keyboard. In the training session, the short and the long standard duration were followed by a 500-ms feedback that took the form of cartoon pictures that varied from trial to trial for the positive feedback, and of a picture of an unhappy Calimero cartoon character for the negative feedback.

### Procedure

#### Bisection task

The participants attended two sessions, one for each duration range. In the 0.5/1-s condition, *S* was equal to 0.5 s and *L* to 1 s. The comparison durations were 0.5, 0.58, 0.67, 0.75, 0.83, 0.92, 1 s. In the 4/8-s condition, *S* and *L* were 4 and 8 s respectively, and the comparison durations were 4, 4.67, 5.33, 6, 6.67, 7.33 and 8 s. In each bisection task, the participants were initially trained to respond short and long after *S* and *L* with the positive/negative feedback following a correct/incorrect response. The participants completed 20 trials with 10 *S* and 10 *L* randomly presented with an inter-trial interval that varied from 0.5 to 2 s. Each trial started with the word “ready!” being displayed on the screen. Then, if the participant was ready, the experimenter pressed the spacebar, and the stimulus duration was displayed after a 500-ms interval. The test phase immediately followed the training phase and the same experimental conditions as in training were used, except that feedback was only given for the anchor durations. Each participant performed 9 blocks of 11 trials each (99 trials): 3 trials for *S* and *L,* and 1 trial for each intermediate duration. In each bisection session, the experimenter told the participants not to count and explained to them that counting time might bias the scientific data [39].

#### Neuropsychological tests

After each bisection session, the participants performed 2 of the 4 neuropsychological tests. The forward and the backward versions of the Corsi Block-tapping test from the non-verbal Wechsler Intelligence Scale [40] were used to assess short-term and working memory capacities, respectively [41]. In this test, the participants recalled the spatial configuration of a block-tapping sequence shown by the experimenter on a board consisting of 9 blocks, either in the same order for the forward Corsi span (short-term memory), or in the reverse order for the backward Corsi span (working memory). To assess selective attention, we used the visual attention subtest of the Developmental Neuropsychological Assessment (NEPSY) [25]. In this test, the participants had to focus selectively and maintain attention on a visual target located in an array of 96 items for a maximum of 3 minutes. The instructions were to find the 20 pictures that were the same as the targets (i.e. cats or faces) in a set of pictures. To evaluate information processing speed, we used the information processing speed test of the Wechsler Intelligence Scales adapted to the age of the participants (WPPSI for the 5-year-olds, WISC for the 8-year-olds and WAIS for the adults) by adding the scores from the coding and the symbol search subtests. In the coding test, the children had to, as quickly as possible and within a period of 2 minutes, either mark a maximum of 65 rows of shapes in accordance with a code (coding test A for children younger than 8 years) or transcribe a maximum of 119 digit-symbols based on another code (coding test B for children older than 8 years and adults). In the symbol search test, the children were given 2 minutes to decide as quickly as possible if a given target symbol appeared in 45 rows of 3 symbols (Searching Test A for children younger than 8 years) or if 1 of 2 different target symbols appeared in 60 rows of 5 symbols (Searching Test B for children older than 8 years and adults). In each row, the target symbol was different. The raw score in the coding test was the number of correct responses, whereas the raw score in the symbol search test was calculated as the number of correct responses minus the number of incorrect responses.

## Results

### Temporal bisection

**Figure 1:**
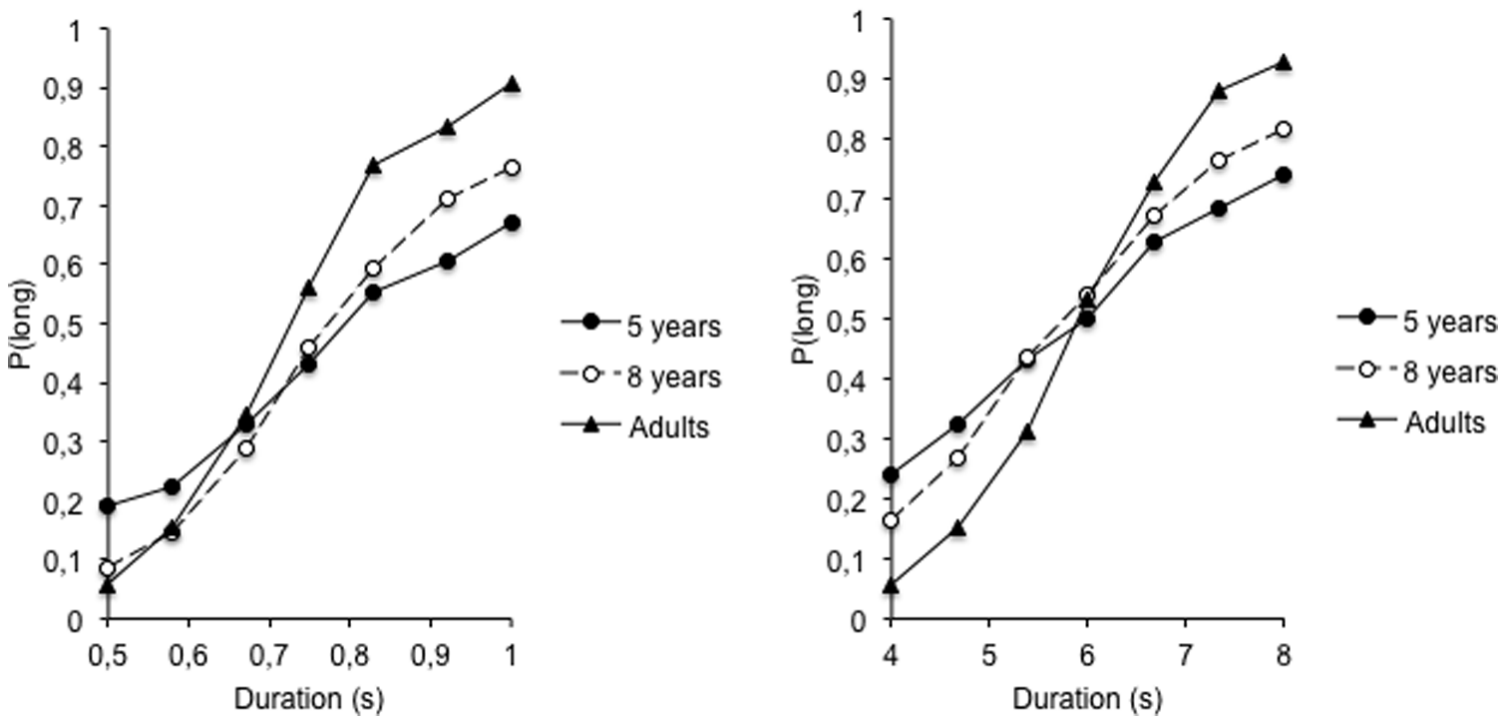
Bisection functions. Proportions of long responses plotted against stimulus durations for the 5-, the 8-year-olds and the adults in the short (0.5/1-s) and the longer (4/8-s) duration range condition.

Figure 1 shows the proportion of long responses (*p*(long)) plotted against the stimulus durations for the 0.5/1-s and the 4/8-s anchor durations. An age-related difference in the psychophysical function can be clearly seen in Figure 1, with the slope of the curves increasing with age. This was confirmed by the analyses of the DL [*D*(*p*(long)  = .75) – *D*(*p*(long)  = .25/2], and the WR [DL/BP]. These measures, as well as that for the BP (*D*(*p*(long)  = .50), were derived from the intercept and slope parameters obtained from the significant fitting of a linear function to the subjects' individual psychophysical functions (5 years: mean *R* ^2^  = .73, *SD*  = .08; 8 years: mean R*^2^*  = .78, *SD*  = .08; Adults: mean *R^2^*  = .87, *SD*  = .04). Table 1 shows the timing measures obtained in this way for each age group for the 0.5/1-s and the 4/8-s anchor durations. The ANOVA on the BP with one within-subject factor (duration) and two between-subject factors (age, gender) showed a significant main effect of duration, *F*(1, 174)  = 3952.79, *p* = .0001, while age, gender and the interaction involving these factors were not significant (all, *p*>.05). Consequently, the BPs for the short duration (M = 0.77, *SE*  = 0.01) were closer to the arithmetic mean [(L+S)/2] (0.5/1-s: 0.75) than to the geometric mean [√(LxS)] (0.5/1-s: 0.71) of the two anchor durations, while the BPs for the long duration were closer to their geometric mean (4/8-s: 5.66) than to their arithmetic mean (4/8-s: 6), and higher in the long than in the short duration condition. Nevertheless, the BP values did not change across the age groups.

**Table 1:** Mean of Bisection Point, Difference Limen and Weber Ratio for the 5-year-olds, the 8 year-olds and the adults in the 0.5/1-s and 4/8-s anchor duration.

	Bisection Point		Difference Limen		Weber Ratio	
	0.5/1-s	4/8-s	0.5/1-s	4/8-s	0.5/1-s	4/8-s
5 years	832	5951	832	5951	0.34	0.38
8 years	783	5814	783	5814	0.22	0.27
Adults	698	5654	698	5654	0.16	0.16

**Figure 2:**
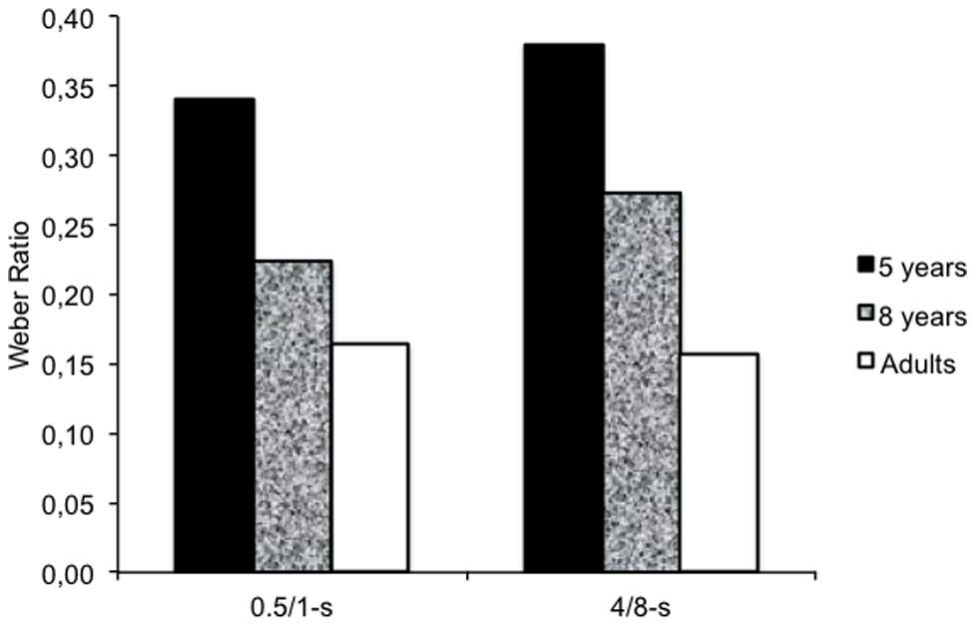
Time sensitivity. Weber ratio for the 5-, the 8-year-olds and the adults in the short (0.5/1-s) and the longer (4/8-s) duration range condition.

In contrast, for the DL and the WR, there was an age effect as illustrated in Figure 2 for the WR. More precisely, for the DL, the ANOVA showed a significant main effect of age, *F*(2, 174)  = 52.21, *p* = .0001, and duration, *F*(1, 174)  = 650.52, *p* = .0001, as well as an age x duration interaction, *F*(2, 174)  = 31.99, *p* = .0001, while the other effects were not significant. This indicated that the variability in time discrimination was higher for the long than for the short durations, but that the magnitude of the difference in DL between the two durations decreased with age, being higher in the 5-year-olds (1.87) than in either the 8-year-olds (1.37) or the adults (0.79), and also higher in the 8-year-olds than in the adults (Scheffé tests, all *p*<.05). For the WR, which is an index of relative temporal sensitivity, only the effect of age reached statistical significance, *F*(2, 174)  = 62.68, *p* = .0001, revealing that the sensitivity to time improved across age groups (Scheffé for all pairwise comparisons, *p*<.05).

The 3-way interaction between age, duration and gender nevertheless just failed to reach significance for the WR, *F*(2, 174)  = 2.89, *p* = .06. This was due to the boys, who obtained a significantly higher WR for the long (4/8-s) than for the short (0.5/1-s) anchor durations at the age of 5, *t*(37)  = 2.31, *p* = .03, and 8 years, *t*(25)  = 2.24, *p* = .03, while the girls obtained the same WR scores for these two duration conditions. At adult age, no difference between the men's and women's WR scores as a function of duration conditions was observed, with the men and women producing similar WRs for the different anchor duration values. This effect of gender will not be further discussed in our manuscript, although other studies have already found a sex effect on temporal performance [42], [43]. It nevertheless suggests that there is a difference in the rhythm at which girls and boys develop time sensitivity for long durations, which are more attentionally demanding, probably as a result of education and/or maturation of the brain [44].

### Correlation between the timing measures and neuropsychological scores

Table 2 shows the means and standard deviations of the scores for the four neuropsychological tests used to assess short-term memory, working memory, selective attention and information processing speed. For each neuropsychological test, there was a significant effect of age, *F*(2, 177)  = 63.38, *F*(2, 177)  = 48.44, *F*(2, 177)  = 47.58, *F*(2, 177)  = 994.04, all *p* = .0001, with all pairwise comparisons being significant (Scheffé, all *p*<.05). Table 3 shows the correlation between the z score for each neuropsychological score, the age and timing measures for which a significant effect of age has been previously found (i.e., for DL in the short and the long duration conditions as a significant duration x age interaction has been found, and for the mean WR as only a main effect of age has been found). The correlations between the scores for the neuropsychological tests and the time sensitivity indexes were significant (all *p*<.001): The higher the working memory, the selective attention and the processing speed scores, the lower the DL and WR values were (indicative of higher sensitivity to time). As there were strong correlations between the different neuropsychological scores, which indicated that there was an overlap between the different dimensions of cognitive control, we ran a hierarchical regression analysis with the scores for each neuropsychological test and age entered into the equation to identify which factor was the best predictor of the variance in each timing measure.

**Table 2:** Scores on the neuropsychological tests for the 5-year-olds, the 8-year-olds and the adults.

	5 years		8 years		Adults	
	M	*SD*	M	*SD*	M	*SD*
Short-Term Memory	5.42	*2.27*	7.60	*1.86*	9.48	*1.77*
Working Memory	3.73	*2.63*	5.52	*1.85*	7.55	*1.79*
Selective Attention	15.93	*8.63*	23.08	*7.82*	29.88	*6.96*
Processing Speed	31.30	*8.64*	59.82	*9.99*	120.88	*14.31*

Abbreviations: M, mean; SD, standard-deviation.

**Table 3:** Correlation between timing measures in bisection, age and z-scores on neuropsychological tests.

	1	2	3	4	5	6	7
1. DL (0.5/1-s)	1						
2. DL (4/8-s)	.42	1					
3. WR	.68	.81	1				
4. Age	−.51	−.53	−.58	1			
5. Short-Term Memory	−.48	−.37	−.48	.56	1		
6. Working Memory	−.47	−.32	−.44	.56	.71	1	
7. Selective Attention	−.36	−.25	−.35	.57	.28	.38	1
8. Processing Speed	−.52	−.56	−.60	.93	.59	.53	.58

**All coefficients significant at 0.01.**

Abbreviations: DL, Difference Limen; WR, Weber Ratio.

*The higher the DL or WR values, the lower the sensitivity to time was.*

As argued in the Introduction, cognitive development may represent a cascade in which changes in processing speed lead to changes in working memory and selective attention capacities. Consequently, we ran an initial hierarchical regression analysis with a specific sequence of causal priority in which processing speed was loaded as the first predictor. Then, the memory variables (short-term and working memory) were entered into equation at the same time, followed by selective attention. Age was added as the last predictor to examine whether it accounted for an additional proportion of variance (beyond that of the other predictor variables) in timing measures. Table 4 summarizes the results of this hierarchical regression analyses for the 3 timing measures: DL (0.5–1-s), DL (4/8-s), and mean WR. However, in order to test our model, we also performed a hierarchical regression analysis with memory scores as the first predictor variable, selective attention scores as the second predictor, and processing speed as the third predictor (Table 5). Age was always included in the last step of the regression analysis. As indicated in Table 4, processing speed explained from 27 to 37% of the variance in timing measures in Model 1 (processing speed loaded as the first predictor). When other variables were added into the analysis, increases in processing speed remained the best predictor of improvement in time sensitivity. Indeed, the scores on this test explained the largest proportion of variance for the DL in the short and the long duration conditions, and for the mean WR in all the models that we employed. The other neuropsychological scores did not emerge as reliable predictors of time sensitivity in temporal bisection, with the exception of the scores in the memory tests in Model 2. Indeed, for WR, processing speed scores were the only reliable predictor across all models. However, for the DL in the short duration condition, adding the memory scores to the information processing speed scores significantly increased the proportion of variance explained. The Δ in the proportion of variance explained nevertheless remained small, although it was relatively greater for the DL in the short duration condition (Δ = .07) than for the WR (Δ = .02).

**Table 4:** Hierarchical regression analyses for the timing measures for the models with the information processing speed as first predictor.

	DL (0.5/1-s)				DL (4/8-s)				WR			
Variables	B	*SE* B	β	*R^2^*	B	*SE* B	β	*R^2^*	B	*SE* B	β	*R^2^*
Model 1												
*1. Processing Speed*	−57.72	7.05	−.52***	.27***	−479.6	52.86	−.56***	.32***	−.08	.01	−.60***	.37***
Model 2												
*1. Processing Speed*	−36.99	8.55	−.34***		−454.1	66.94	−.53***		−.06	.01	−.48***	
*2. Short-Term Memory*	−17.68	10.32	−.16		−42.06	80.84	−.05		−.02	.01	−.13	
*2. Working Memory*	−19.63	9.79	−.18*		−1.44	76.67	−.01*		−.01	.01	−.10	
*Overall significance*				.34***				.*32*				.*39* *
Model 3												
*1. Processing Speed*	−31.81	9.92	−.29**		−514.3	77.41	−.60***		−.06	.01	−.48***	
*2. Short-Term Memory*	−19.83	10.53	−.18		−17.11	82.16	−.02		−.02	.01	−.13	
*2. Working Memory*	−17.46	10.02	−.16		−26.66	78.13	−.03		−.01	.01	−.10	
*3. Selective Attention*	−8.77	8.53	−.08		101.95	66.51	.12		.01	.01	−.01	
*Overall significance*				.*34*				.*33*				.*39*
Model 4												
*1. Processing Speed*	−25.41	19.30	−.23		−439.4	150.45	−.52**		−.05	.02	−.39*	
*2. Short-Term Memory*	−20.24	10.61	−.18		−21.99	82.74	−.03		−.02	.01	−.13	
*2. Working Memory*	−16.54	10.32	−.15		−15.85	80.46	−.02		−.01	.01	−.09	
*3. Selective Attention*	−8.59	8.56	−.08		104.0	66.72	.12		.01	.01	−.01	
*4. Age*	−.07	.19	−.07		−.87	1.50	−.10		.01	.01	−.10	
*Overall significance*				.*35*				.*33*				.*39*

Abbreviations: DL, Difference Limen; WR, Weber Ratio; B, Unstandardized beta coefficient; SE B, Standard error on beta; β, Standardized beta coefficient.

* *p*<.05; ** *p*<.001; *** *p*<.001.

**Table 5:** Hierarchical regression analyses for the timing measures for the models with the memory (short-term and working memory) as first predictor.

	DL (0.5/1-s)					DL (4/8-s)					WR	
Variables	B	*SE* B	β	*R^2^*	B	*SE* B	β	*R^2^*	B	*SE* B	β	*R^2^*
Model 1												
*1. Short-Term Memory*	−33.90	10.09	−.31**		−241.2	84.36	−.28**		−.04	.01	−.34***	
*1. Working Memory*	−27.53	10.09	−.25**		−98.46	84.36	−.12		−.03	.01	−.20*	
*Overall significance*				.27***				.14***				.25***
*Model 2*												
*1. Short-Term Memory*	−33.57	9.87	−.30**		−239.4	83.73	−.28**		−.04	.01	−.33***	
*1. Working Memory*	−19.07	10.26	−.17		−52.69	87.07	−.06		−.02	.01	−.12	
*2. Selective Attention*	−22.65	7.53	−.21**		−122.5	63.93	−.14		−.03	.01	−.21**	
*Overall significance*				.30**				.*16*				.29**
Model 3												
*1. Short-Term Memory*	−19.83	10.53	−.18		−17.11	82.16	−.02		−.02	.01	−.13	
*1. Working Memory*	−17.46	10.02	−.16		−26.66	78.13	−.03		−.01	.01	−.10	
*2. Selective Attention*	−8.77	8.53	−.08		101.95	66.51	.12		.01	.01	−.01	
*3. Processing Speed*	−31.81	9.92	−.29**		−514.3	77.41	−.60***		−.06	.01	−.48***	
*Overall significance*				.34**				.33***				.39***
Model 4												
*1. Short-Term Memory*	−20.24	10.61	−.18		−21.99	82.74	−.03		−.02	.01	−.13	
*1. Working Memory*	−16.54	10.32	−.15		−15.85	80.46	−.02		−.01	.01	−.09	
*2. Selective Attention*	−8.59	8.56	−.08		104.00	66.72	.12		.01	.01	−.01	
*3. Processing Speed*	−25.41	19.30	−.23		−439.4	150.45	−.52**		−.05	.02	−.39*	
*4. Age*	−.07	.19	−.07		−.87	1.50	−.10		.01	.01	−.10	
*Overall significance*				.*34*				.*33*				.*39*

Abbreviations: DL, Difference Limen; WR, Weber Ratio; B, Unstandardized beta coefficient; SE B, Standard error on beta; β, Standardized beta coefficient.

* *p*<.05; ** *p*<.001; *** *p*<.001.

**Figure 3:**
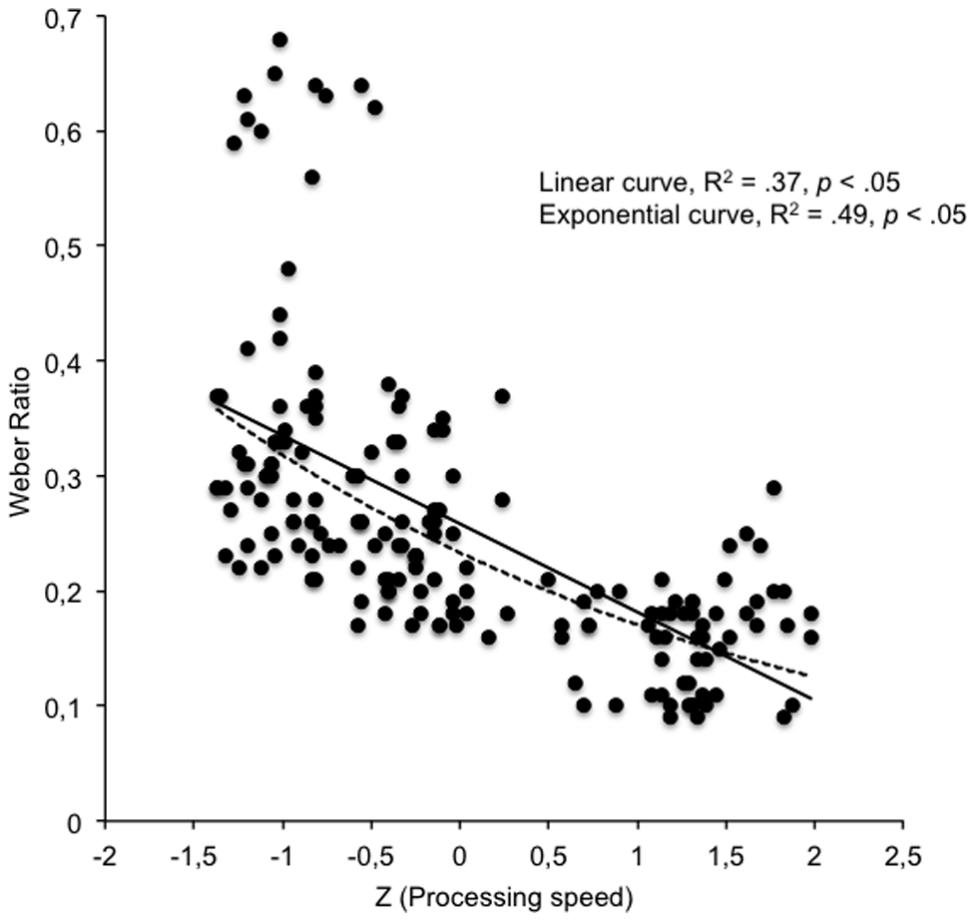
Time sensitivity and information processing speed. Significant correlation between Weber Ratio and information processing speed (z-scores).

As indicated in Table 5, Model 1 (memory scores loaded as the first predictor) explained 27 %, 14% and 25% of variance in the DL (0.5/1-s), the DL (4/8-s) and the WR values, respectively. When selective attention scores were added to the memory scores in the Model 2, there was a slight increase in the proportion of variance explained (Δ *R* ^2^  = .03 for DL (0.5/1-s); Δ *R* ^2^  = .04 for WR). However, once processing speed was entered into equation, it was the only predictor variable that accounted for the variance in every temporal sensitivity index (DL (0.5/1-s), *ß* = .29; DL (4/8-s), *ß* = .60; WR, *ß* = .48, all *p*<.05). As revealed by Model 4, age did not increase the proportion of variance explained. In sum, our hierarchical regression analyses demonstrated that processing speed was the major predictor of variances in age-related differences in time sensitivity. Figure 3 illustrates this strong relationship between the WR value and the information processing speed scores, which took the form of a linear regression function or, more accurately, an exponential decay function (*R* ^2^  = .49, *p*<.05): The higher the information processing scores, the lower the WR values were. In other words, time sensitivity increases when information processing speeds up as children grow older.

As discussed below, time processing is therefore an inherent part of the speed of execution of cognitive tasks such as those tested in the subtests of the Wechsler Intelligence Scale for Children that assess information processing speed. Therefore, and in line with Rammsayer and Brandler, we decided to examine whether, conversely, sensitivity to time was a good predictor of variance in scores in the information processing speed test [45]. Consequently, we entered an index of time sensitivity (mean WR) in the first step of the hierarchical regression analysis, and the scores from the other neuropsychological tests, i.e., the short-term and the working memory scores in the second step and the selective attention scores in the last step of this regression procedure (Table 6). The overall *R* ^2^ for Model 1 was significant (*p*<.0001) and revealed that the WR took account for 37% of variance in the processing speed scores. However, the proportion of variance explained increased when the scores on the memory tests (Model 2, Δ *R* ^2^  = .12) and those on the selective attention test were added to the time sensitivity index (Model 3, Δ *R* ^2^  = .11). In fact, the summary of the hierarchical regression models revealed that time sensitivity, selective attention and short-term memory were all reliable predictors of individual differences in processing speed (all *p*<.01) that together accounted for 60% of the variance.

**Table 6:** Hierarchical regression analyses for the information processing speed scores.

Variables	B	*SE* B	β	*R^2^*
Model 1				
*1. WR*	−4.75	.47	−.60***	.37***
Model 2				
*1. WR*	−3.14	.49	−.40***	
*2. Short-Term Memory*	.31	.08	.31***	
*2. Working Memory*	.13	.07	.13	
*Overall significance*				.49***
Model 3				
*1. WR*	−2.45	.44	−.31**	
*2. Short-Term Memory*	.33	.07	.33***	
*2. Working Memory*	.01	.07	.01	
*3. Selective Attention*	.37	.05	.37***	
*Overall significance*				.60***

Abbreviations: WR, Weber Ratio; B, Unstandardized beta coefficient; SE B, Standard error on beta; β, Standardized beta coefficient.

* *p*<.05; ** *p*<.001; *** *p*<.001.

## Discussion

In line with the findings of many developmental studies, our results found that the scores on neuropsychological tests assessing short-term memory, working memory, selective attention, and information processing speed increased with age until early adulthood. In addition, there was a large overlap between the cognitive processes assessed by these different tests, as the positive correlation between the scores on the different neuropsychological tests revealed. Nevertheless, as Luna et al. have argued, although these cognitive processes interact, each of them makes a specific contribution to the control of cognition [37]. As far as the discrimination of time is concerned, previous studies have suggested that the development of selective and sustained attention assessed by the attention/concentration index of the Children's Memory Scale (CMS) [46] accounts for individual differences in temporal sensitivity for long durations (>15 s) [22], while the development of selective attention accounts for these differences in temporal sensitivity to the difficult ratio of 2:3 between the two anchor durations [24]. However, in time discrimination tasks such as that used in the present study, in which the ratio between *S* and *L* is 1 2, working memory capacity has been identified as the best predictor of improvement in time sensitivity for the short (<1 s) and the long durations (from 4 to 8 s) [22], [23]. The results of the present study thus confirm the important role played by the development of working memory capacities in the improvement of temporal discrimination and therefore demonstrate that the temporal judgment tasks used in humans impose demands at the level of controlled processes. However, our results also suggest that working memory capacity is not sufficient in itself to explain age-related changes in time sensitivity in a temporal discrimination task. Indeed, our current findings show that information processing speed accounts for a larger proportion of individual variance in children's time sensitivity than working memory.

While only very few neuropsychological studies of time discrimination have been conducted in healthy children, several studies have examined the relationships between scores on different neuropsychological tests and time judgments in human adults. The studies conducted in healthy adults have effectively highlighted the critical role of working memory in timing [47]–[49]. For instance, Broadway and Engle recently showed that individuals with high working memory capacities reproduced durations more accurately and with less variability than those with lower working memory capacities [47]. Studies comparing young and elderly subjects have also shown that deficits in working memory capacities can help explain deficits in time judgments with aging [50]–[53]. This finding is consistent with the results of studies that have used an interference task paradigm to show that temporal performance decreases when working-memory demands increase [54]–[56]. All authors have therefore concluded that maintaining and capturing the flow of temporal information in a temporal task require working memory capacities. However, in these studies, when processing speed was factored out (simple reaction time, temporal rhythm), working memory explained a large proportion of individual variances in temporal accuracy in temporal reproduction tasks but not in temporal production tasks [50], [51], [52]. Therefore, in a production task, working memory was not found to be a predictor of variance in temporal accuracy [51], [52]. The fact that information processing speed is a better predictor of temporal accuracy than working memory is entirely consistent with our findings in children in a temporal bisection task (although our data related to temporal variability and not to temporal accuracy). The cognitive processes involved in working memory overlap those that contribute to information processing speed. However, this later is itself characterized by cognitive dimensions other than the storage and maintenance of information in memory, namely the speed-dependent efficiency of information processing [57]. Indeed, the cognitive tasks involved in tests that assess information processing speed have to be performed under temporal constraints, i.e. as quickly as possible. Consequently, processing speed and timing are interdependent. In our study, the test scores for processing speed were the best predictor of developmental changes in time sensitivity in a bisection task and, conversely, sensitivity to time was the best predictor of developmental changes in the scores in the information processing speed tests, although in this latter test, attention and memory capacities are also required.

Rammsayer and colleagues, who obtained similar results in human adults, hypothesized “a temporal resolution power” and suggested that the capacity for temporal accuracy would be a major predictor of general intelligence (factor g) [45], [58], [59]. As these authors argued, the degree of temporal resolution would be an indicator of the dynamic physiological activity of our brains: A higher rate of neuronal oscillation should bring about faster and more efficient information processing and a higher level of temporal resolution. Several models of timing have been proposed based on mechanisms involving neural oscillations [60], [61]. Nevertheless, the most popular of these models in the neuroscientific field is the Striatal Beat Frequency (SBF) model proposed by Mattell and Meck [62], [63]. According to this model, the representation of time emerges from synchronous neural oscillations occurring across distributed cortical regions at the moment of the stimulus to be timed. In addition, the spiny neurons in the striatum, which are active at certain critical times, are thought to play the role of coincidence detectors. As training progresses, these neurons fire when they recognize a pattern of coincidences of oscillators associated with the stimulus that is to be timed. Within this framework, it seems likely that the speeding up of information processing with age indirectly reflects the increasing dynamic efficiency of the neural circuits responsible for timing as the brain matures. The role of neural oscillators in the perception of time has found support in Treisman et al. 's research showing that periodic clicks influence time judgments as a function of their rate by affecting the frequency of cortical oscillations [61]. More than 40 years ago, Survillo also established a link between neural oscillations and information processing speed (reaction time): the faster the alpha rhythm, the faster information processing is [64], [65]. As far as electrical brain activity in children is concerned, EEG activity changes from birth through to adolescence, with alpha frequency increasing (8–12 Hz) and theta frequency decreasing (4–8 Hz) ([66], [67], [68] for a review). These age-related changes in EEG are thought to reflect the continuing maturation of neural circuits (e.g., myelination). They might therefore indicate the age-related acceleration in neural oscillatory activities that underpin time representation. As stated by Rammsayer and Brandler, the higher the frequency of neural oscillators, the finer the temporal resolution of the internal clock [58]. In addition, brain maturation may be accompanied by an improvement in the synchronization between the neuronal groups, on the one hand, and, on the other, in the connection between cortical and striatal structures due to neural differentiation and an increase in axonal conduction velocity [19]. EEG coherence is a measure of the degree of correlation between different EEG sites and provides information about the neurophysiological dynamics of the maturing brain [19]. More specifically, the coherence distance increases with age up to 5 years, especially in the posterior-anterior direction, thus suggesting that differentiated subsystems may become integrated. Changes continue to occur at the ages of approximately 9 and 14 years but, in this case, from long to shorter distances and reflect a process involving the differentiation of integrated subsystems [19]. This is consistent with fMRI studies indicating that areas activated in cognitive tasks in children are both more local in the brain, but also larger and more “diffuse”. In sum, the age changes in information processing speed may be an indirect measure that reflects the maturation of the functioning of cerebral systems that underpin the processing of time. However, little neurodevelopmental research has been devoted to the performance of temporal tasks in typically developing children, with the exception of the two recent fMRI studies conducted by Smith et al. and Neufang et al. among participants aged from 10 to 53 years and from 8 to 15 years, respectively [69], [70]. In their fMRI study, Smith et al. observed a progressive increase with age in the activation of both the right dorsolateral striatum and the left dorsolateral prefrontal and parietal regions [69]. Further investigations of children's time perception using measures of the dynamic activity of the brain are thus required.

In conclusion, the mechanisms underlying the processing of time seem to speed up during development. In other words, as development progresses, the timing mechanisms run faster and temporal precision improves. As Buhusi and Meck point out, the human brain is a time machine [71]. Rammsayer and Brandler used the metaphor of “internal master clock” [58]. We can now go further and say that the temporal resolution of this master clock improves with development. However the acceleration of this master clock affects a complex cascade of cognitive processes (working memory, attention) that improves not only the efficiency of temporal information processing, but also the processing of all dynamic information that, by definition, unfolds in time [72], [73]. Indeed, time is inherent to the dynamic physiological activity of our brain, and it is not therefore surprising that the development of timing capacities originates in basic mechanisms that accelerate the general dynamic functioning of the brain and which, in turn, affect other related cognitive processes.
